# Probing the overarching continuum theory: data-driven phenotypic clustering of children with ASD or ADHD

**DOI:** 10.1007/s00787-022-01986-9

**Published:** 2022-06-10

**Authors:** M. K. Deserno, J. Bathelt, A. P. Groenman, H. M. Geurts

**Affiliations:** 1 Dutch Autism and ADHD Research Centre (d’Arc), Department of Psychology, University of Amsterdam, Amsterdam, The Netherlands; 2 Leo Kannerhuis, Amsterdam (Youz, Parnassiagroep), Amsterdam, The Netherlands; 3 Max Planck Institute for Human Development, Berlin, Germany; 4 Royal Holloway, University of London, Egham, UK

**Keywords:** ADHD, Autism, Psychiatric nosology, Overarching continuum theory, Data-driven clustering

## Abstract

The clinical validity of the distinction between ADHD and ASD is a longstanding discussion. Recent advances in the realm of data-driven analytic techniques now enable us to formally investigate theories aiming to explain the frequent co-occurrence of these neurodevelopmental conditions. In this study, we probe different theoretical positions by means of a pre-registered integrative approach of novel classification, subgrouping, and taxometric techniques in a representative sample (*N* = 434), and replicate the results in an independent sample (*N* = 219) of children (ADHD, ASD, and typically developing) aged 7–14 years. First, Random Forest Classification could predict diagnostic groups based on questionnaire data with limited accuracy—suggesting some remaining overlap in behavioral symptoms between them. Second, community detection identified four distinct groups, but none of them showed a symptom profile clearly related to either ADHD or ASD in neither the original sample nor the replication sample. Third, taxometric analyses showed evidence for a categorical distinction between ASD and typically developing children, a dimensional characterization of the difference between ADHD and typically developing children, and mixed results for the distinction between the diagnostic groups. We present a novel framework of cutting-edge statistical techniques which represent recent advances in both the models and the data used for research in psychiatric nosology. Our results suggest that ASD and ADHD cannot be unambiguously characterized as either two separate clinical entities or opposite ends of a spectrum, and highlight the need to study ADHD and ASD traits in tandem.

**Supplementary Information:**

The online version contains supplementary material available at 10.1007/s00787-022-01986-9.

## Introduction

The prevalent co-occurrence of Autism Spectrum Disorder (ASD) and Attention-Deficit Hyperactivity Disorder (ADHD) reflects a pressing problem for diagnosis and treatment in child psychiatry [58, 69, 75]. The two diagnostic categories share etiological factors and overlapping behavioral characteristics (e.g., symptoms of inattention and impulsivity [70]). Common practices of small sample size studies and case–control models, however, have stalled progress in the pursuit of a better understanding of the discriminant properties between these two neurodevelopmental conditions. Here, we employ a data-driven clustering approach to investigate whether these neurodevelopmental conditions comprise of subtypes that cross clinical boundaries in a large cohort of atypically and typically developing children, and cross-validate our results subsequently.

A growing body of literature is concerned with the diagnostic validity of classification based on expert consensus [40], such as the International Disease Classification [ICD] [83] and the Diagnostic and Statistical Manual of Mental Disorders [DSM] [3]. Using DSM-5 criteria, Attention-Deficit Hyperactivity Disorder (ADHD) is the most commonly diagnosed mental condition in children with a lifetime prevalence between 5 and 7%. ADHD is characterized by inattention, hyperactivity, and impulsivity, which encompass difficulties to stay on task, sit still, and wait for one’s turn. In contrast, autism spectrum disorder (ASD) is considered to be a rarer condition with a prevalence of around 2%. The behavioral characteristics of ASD are difficulties with social communication and interaction alongside so-called restricted, repetitive, and/or stereotyped behaviors and narrow interests (RRBIs). Despite these seemingly different behavioral presentations, clinical experts and researchers have long recognized that there is considerable overlap between ADHD and ASD. Formal comparisons indicated that between 20 and 80% of children with a diagnosis of ASD also meet DSM-IV criteria for ADHD. In fact, ADHD is the most common comorbid problem in children with ASD. In turn, 30–60% of those with ADHD showed clinically significant symptoms of ASD. Notably, these problems encompass all characteristics of ASD, including difficulties with social interaction/communication and RRBIs. This overlap has been recognized in the latest revision of the Diagnostic and Statistical Manual of Mental Disorder (DSM). While ADHD and ASD were mutually exclusive diagnoses in previous iterations of the DSM, DSM-5 allows for comorbid ADHD and ASD diagnoses.

Recent work hypothesizes that ADHD and ASD should not be conceptualized as distinct neurodevelopmental conditions but rather as manifestations of one overarching condition with a similar etiology [34, 42, 80]. The hypothesis underlying this theory considers ASD to be a manifestation of the most “severe” subtype on one end of the overarching continuum, while mild ADHD would be located on the other end of this hypothesized continuum. If this hypothesis holds, its theoretical implication would be that ASD cannot exist without ADHD. One would, therefore, expect the categorical classification of individuals as either ASD or ADHD cases to be difficult, since there is a sliding scale between the symptoms rather than two distinct clinical entities. Genetic studies indicate considerable overlap between ADHD and ASD, but also some differences. Regarding inheritance, a large population study indicated a higher probability to be diagnosed with ADHD for relatives of individuals with ASD, which was substantially higher for monozygotic than dizygotic twins [28]. This overlap was explained by genetic effects [29]. Furthermore, a large number of known copy-number variants (CNVs) and chromosomal abnormalities are associated with a higher likelihood for ADHD and ASD, including 2q11.2 deletions, and duplications of X and Y chromosomes among others (see for [4] a recent review). A cross-syndrome genome-wide association study (GWAS) also indicated a shared risk for ADHD and ASD [20]. However, there may be partially distinct genetic influences as indicated by a recent study that reported a weak negative correlation between genetic factors associated with ADHD and ASD [12].

A parallel line of reasoning suggests equifinality in relation to atypical attention which may stem from inhibitory atypicalities when it comes to ADHD but social atypicalities in the case of ASD phenotypes [39, 82]. This interpretation is supported by several studies that have shown condition-specific effects for the same psychiatric drugs, such as different effects of selective serotonin-reuptake inhibitors (SSRI) in ADHD and ASD [14]. One such SSRI has been shown to improve inattentiveness in ADHD [66] through upregulating decreased frontal activation, and improve social interaction and stereotypies in ASD by downregulating increased frontal activation [37].

Difficulties in executive function have been identified in both ADHD and ASD in childhood and in adult samples [27, 33, 62]. While both conditions show difficulties with multiple aspects of executive function, the executive dysfunction profiles may be partially distinct in ADHD and ASD. Children with ASD were found to show more problems with flexibility and planning, while children with ADHD had more difficulties with inhibition [27, 33, 63]. Difficulties in social cognition have also been identified in both ADHD and ASD. Difficulties in emotion recognition and theory of mind have been identified in both ADHD and ASD, but the difficulties seem milder in ADHD and are not present in adults with ADHD [10].

Regarding brain-level overlap, studies that investigated the neural basis of ADHD and ASD similarly suggest similarities in the brain systems involved. Both conditions are increasingly characterized as conditions related to atypical brain connectivity involving differences in the interplay between large-scale brain systems rather than arising from focal differences in specific brain areas. The most prominent differences in ADHD have been observed in the integration of the default mode network (DMN), the salience network (SN), the ventral attention network (VAN), and the frontoparietal attention network (FAN, [19, 76]). Furthermore, the basal ganglia, particularly frontostriatal circuits, have been consistently implicated in ADHD [22, 26]; ). The most consistent differences in connectivity in ASD have been identified in the DMN [6, 8, 30, 57, 64]. Some studies also reported hypoconnectivity in the SN [38] and connections between the medial and orbital prefrontal cortex (PFC) with the amygdala [31]. Three studies examined similarities and differences in ADHD and ASD in functional brain organization. Kernbach and colleagues identified brain-level phenotypes in the connectivity of the DMN, SN, and dorsal attention network (DAN) in ADHD and ASD. The results indicated that ADHD was characterized by reduced connectivity between the DMN and DAN, and ASD by reduced DMN-SN connectivity [46]. Di Martino et al. identified hypoconnectivity in the precuneus that is common to both ADHD and ASD, and showed that connectivity of the basal ganglia distinguished ASD participants with or without comorbid ADHD symptoms [21]. Bathelt et al. showed that machine learning classification based on resting-state brain connectivity can distinguish ASD and ADHD participants from controls, but performs at chance level when attempting to distinguish ASD and ADHD cases [6].

In the current study, we tested different explanations for the overlap between ASD and ADHD by exploring (1) whether the overlap of the diagnostic groups stems from undistinguishable behavioral profiles, (2) whether their overlap arises from overlapping subgroups (e.g., combined ASD-ADHD), or (3) whether their overlap arises from an underlying behavioral continuum. In a first step, using random forest classification, we investigated whether participant scores on the behavioral measures (Strengths and Weaknesses of ADHD symptoms and Normal-behaviors ratings scale [SWAN], Social Responsiveness Scale [SRS]) have sufficient discriminatory power to assign participants to diagnostic groups. Based on these results, we then employed a community detection approach to identify potential subtypes across the ADHD-ASD spectrum. By employing taxometric analyses, we then tested whether the classification performance may arise because of an underlying continuum.

## Methods

### Participants

The current analysis was based on the existing data obtained from the Child Mind Institute Biobank database (https://childmind.org, date of access: February 21st, 2019). The initial sample consisted of 475 children (ADHD: 249, ASD: 90, typically developing [TD]: 136) between 7 and 13 years of age. This sample is part of a larger cohort of the Healthy Brain Network Biobank based on a community-referred recruitment model of children with developmental psychopathology (see [1] for more details). One participant in the ASD group and eight participants in the TD group were removed because of missing questionnaire data or missing diagnostic information. Diagnostic classifications were based on extensive clinicians-administered assessments, including the Autism Diagnostic Observation Schedule (ADOS) for suspected autism [1]. All participants in the sample were first administered a computerized web-based semi-structured DSM-5-based psychiatric interview (parent interview and child interview used to derive clinical diagnoses by a licensed clinician). This computerized interview was a web-based version of the Schedule for Affective Disorders and Schizophrenia—Children’s Version (KSADS-COMP [45]), and resulted in an automated diagnosis. Upon completion of the interviews and review of all materials, clinically synthesized diagnoses were generated by the clinical team [1]. The classification reported in the current study was based on these consensus clinical diagnoses. In addition, we used structured questionnaire data from the self-administered assessment protocol entered through the online patient portal [1]. To detect and remove potentially careless responses, we calculated the Mahalanobis distance [84]. According to this measure, 32 participants fell outside of the recommended distance threshold and were removed from the analysis (Mahalanobis distance > 14.07,18 ADHD, 12 ASD, 2 TD). We also tested for univariate outliers, defined as data points that fell more than 3 standard deviations above or below the mean of the sample on any scale. Only one datapoint met this criterion. This datapoint also met the multivariate outlier criterion. The final sample consisted of 434 children (231 ADHD, 77 ASD, and 126 TD). There were no significant differences in age between the diagnostic groups, but there was a disproportionate number of boys in the ADHD and ASD groups (see Table 1) consistent with the greater prevalence of these diagnoses in males [54]. A third of the children in the ADHD group had an additional diagnosis. The most common were Oppositional Defiant Disorder (*n* = 72 [29.38%]), Autism Spectrum Disorder (*n* = 39 [15.92%]), Specific Learning Disorder with Impairment in Reading (*n* = 34 [13.88%]), Language Disorder (*n* = 33 [13.47%]), Generalized Anxiety Disorder (*n* = 23 [9.39%]), and other less frequent diagnoses (e.g., Enuresis, Specific Phobias, Separation Anxiety; *n* < 20 [≦5%]^1^). Around a fifth of children in the ASD group had an additional diagnosis. The most common diagnoses were ADHD-Combined Type (combination of hyperactive and inattentive symptoms [DSM-5], *n* = 36 [14.69%]), ADHD-Inattentive Type (*n* = 13 [5.31%]), and other less frequent diagnoses (e.g., Oppositional Defiant Disorder, Specific Learning Impairment, Generalized Anxiety Disorder; *n* < 10 [≦ 5%]).

**Table 1:** Comparison of demographic information between the diagnostic groups

Age [year]	Mean ± SE	Comparison	
ADHD	9.4 ± 1.7	ADHD vs. ASD	*t* (131) = 0.58, *p* = 0.566
ASD	9.3 ± 1.6	ADHD vs. TD	*t* (273) = -0.13, *p* = 0.899
TD	9.4 ± 1.5	ASD vs. TD	*t* (153) = -0.63, *p* = 0.527

Sex [male]	*n* (%)		
ADHD	186 (80.5)	**ADHD vs. even**	***Χ***^**2**^** (1) = 86.06, *p* < 0.001**
ASD	65 (84.4)	**ASD vs. even**	***Χ***^**2**^** (1) = 36.48, *p* < 0.001**
TD	58 (46.0)	TD vs. even	Χ^2^ (1) = 0.79, *p* = 0.373

Sex ratio was compared to an equal split between male and female participants (even). Bold text indicated significant results at *p* < 0.05

### Pre-registration

**Fig. 1:**
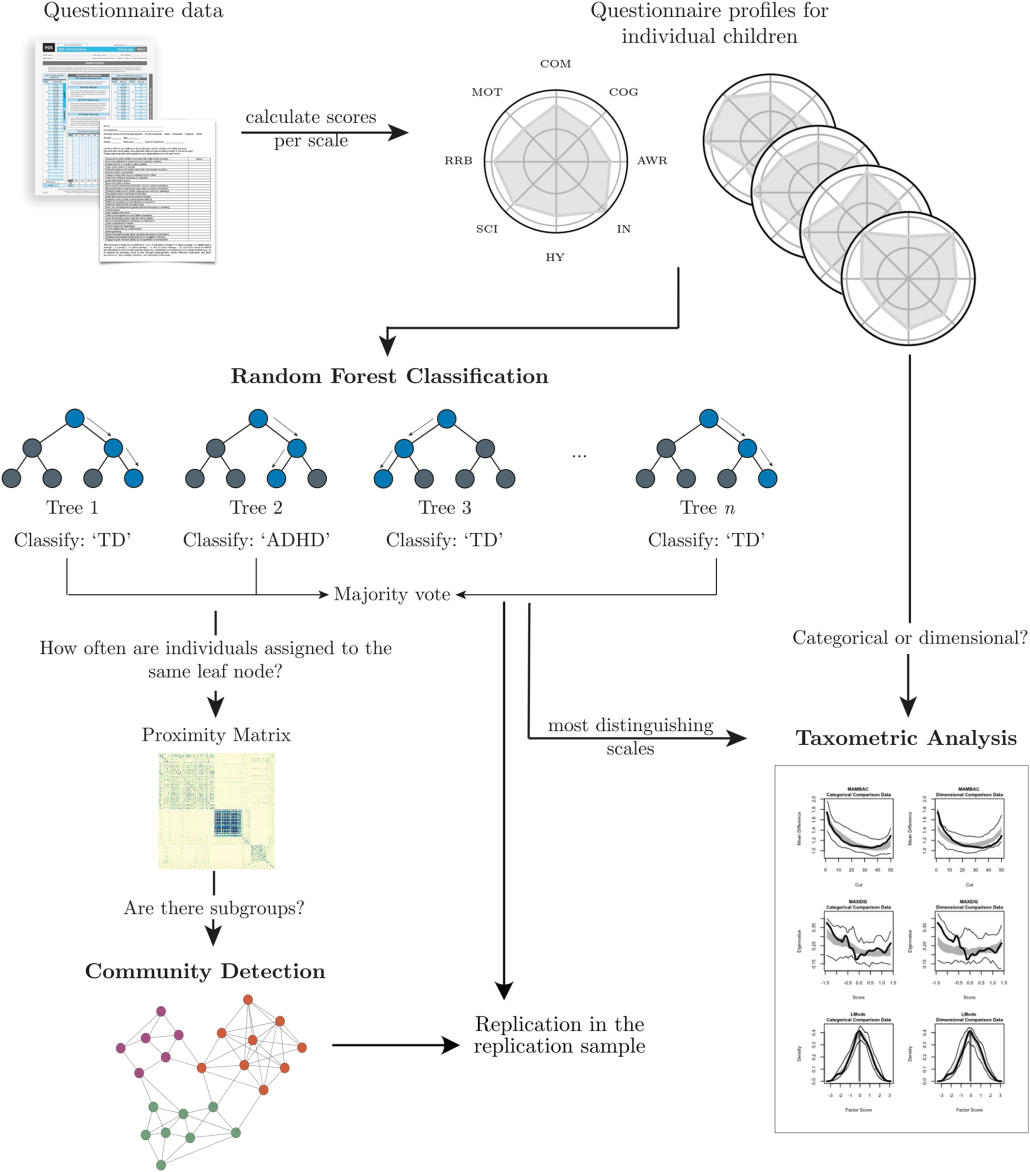
Overview of the analysis steps. First, item scores were summarized within questionnaire scales to obtain individual profiles. Then, the profiles were used to predict the diagnostic labels using random forest classification. The proximity matrix generated by the random forest classification was used to detect subtypes. The community detection and random forest classification were tested in the replication sample. In addition, the questionnaire scales that best distinguished the diagnostic groups were used for taxometric analysis to determine if a categorical or a dimensional account provided a better fit to the data

The analysis steps (see also Fig. 1) and expected results were pre-registered before accessing the data. The pre-registration can be accessed online (https://aspredicted.org/ya7wr.pdf).

### Analysis code

The code for the analyses is available via the Open Science Framework (https://osf.io/vkwma/?view_only=1e66771d9b8c4f1dab7af35918345432).

### Materials

The Strengths and Weaknesses of ADHD symptoms and Normal-behaviors ratings scale (SWAN; Swanson et al., 2001) is a questionnaire with 18 items that assesses potential strengths and weaknesses related to ADHD symptoms on a single parent-rated scale. It uses items from the Swanson Nolan And Pelham IV (SNAP-IV; [78], but the SWAN rephrases the symptoms into strength-based statements making them follow a normal distribution instead of a skewed distribution [2]. The SWAN items are grouped into the Hyperactivity/Impulsivity (HY) and the Inattention (IN) subscale. A validation study of the SWAN indicated high internal consistent (Cronbach’s alpha = 0.95 and adequate test–retest reliability *r* = 0.66, [51]).

The Social Responsiveness Scale (SRS; [17]) is a 65-item scale that is designed to obtain parent- or teacher-ratings of autistic symptomatology as observed in naturalistic social settings. The SRS-assessed symptoms are combined into five subscales: Social Awareness (AWR), Social Cognition (COG), Social Communication (COM), Social Motivation (MOT), and Restricted Interests and Repetitive Behaviors (RRB). Validation studies have shown that the SRS has good psychometric properties (3-month test–retest reliability: 0.88, inter-rated reliability: 0.8, correlation with the Autism Diagnostic Interview Revised (ADI-R) score: 0.7; [16, 17]). Of note to the current investigation is that although the SRS was originally designed to produce continuously distributed scores, recent results indicated a bimodal distribution in family members of children with ASD that distinguishes family members who meet ASD criteria from those who do not [18, 81].

After careful consideration of how to best match the different behavioral instruments used in the different samples, we decided not to use the Disruptive Behavior Disorders Rating Scale from the CMI data. We therefore not fully followed our pre-registered analyses plan, and focused solely on the SRS and SWAN data for the purpose of replication with the independent replication sample.

*Replication sample* The independent replication sample consisted of 219 children (73 female, ADHD: 87 [39.73%], ASD: 69 [31.51%], TD: 63 [28.77%]) between 8 and 12 years (mean: 10.11, SE: 0.092). For the purpose of replication, we focus solely on the SWAN and SRS data. The replication sample was from the Oregon ADHD and Autism project and included community recruited volunteers with ADHD and/or ASD diagnosis confirmed by multi-method, multi-measure, best estimate procedure based on the DSM-IV. For detailed description of this sample, see Karalunas et al. [44]. This dataset was used as a replication sample to ascertain if the results from the main sample generalize to other samples that are more similar to community diagnosis of ADHD and ASD.

### Random forest classification

First, we applied random forest classification (RFC) to investigate if the selected questionnaire scales can be used to classify participants into diagnostic groups (ADHD, ASD, and TD). For multi-class classification, the diagnostic groups were recoded according to a one-versus-all coding scheme, e.g., ADHD vs. ASD and TD. The RFC model was tuned and trained in a random subsample of 75% of the participants and 25% of the data were held-out for the final validation (outer-loop). To identify the optimal tree depth (i.e., the more splits the more detailed information is explained), bootstrap cross-validation with 10,000 random resamples was employed (tree-depth tuning range: 1 to total number of scales). Synthetic minority oversampling (SMOTE) was used to account for class imbalance in the subsets [15]. This method creates synthetic data points that are nearest neighbors to the observed data points in feature space. The area under the receiver-operating characteristic curve (AUROC) averaged across all classes was used to tune the model. These procedures were implemented in R v3.5.2 using the *randomForest* v4.6 [53] and *caret* v.6.0 [49] packages. To work with the best performing classification approach, we evaluated and compared the classification performance of alternative machine learning approaches (l1-/l2-regularized support vector classification, ridge regression) and cross-validation strategies (k-fold, stratified shuffle split). The machine learning approach presented in the main analysis showed better or equivalent performance as these alternatives (the detailed results are included in the Supplementary Materials). The full analysis code is available online (https://osf.io/vkwma/).

### Community detection

To investigate if there are subtypes that may explain the overlap between ADHD and ASD, we employed a community detection approach based on the clinically sensitive questionnaire scales from the RFC. Community detection is an optimization clustering method to detect communities, or subgroups, of nodes (e.g., people), within networks. In the current analysis, the network is based on the RFC proximity matrix which represents the proximity of each participant to all other participants in the sample according to the RFC solution. The proximity indicates how often two participants were assigned to the same leaf node across decision trees in the random forest that aimed to predict the diagnostic label using splits on the questionnaire ratings. The advantage of applying the community detection to the proximity matrix is that the subgroups are necessarily relevant to the diagnostic categorisation [23], whereas grouping based on, e.g., the correlation of questionnaire scales, may be influenced by other characteristics, such as variance of the scale.

Here, the Louvain algorithm was used for community detection [9] followed by a fine-tuning step using the Kernighan-Lin algorithm [47]. Due to randomness in the initial assignment of nodes to communities, the algorithm may produce slightly different results at different instantiations. To reach a stable assignment, the algorithm was run 100 times to construct an agreement matrix, which was then used to obtain a consensus community partition [52]. We repeated this procedure for multiple resolutions (varying γ between 0.1 and 5.0, [67]). We selected the solution that provided the best separation and internal consistency of groups (maximal modularity index) while providing the highest agreement across different resolutions (maximal normalized mutual information) between successive values of γ. The best solution was indicated at γ = 0.2. To estimate the reliability of the clustering at this value of γ, we repeated the clustering with randomly selected subsets of 80% of the data and compared the results to clustering with the full dataset across 100 repetitions [79]. The results indicated high stability of the clustering (mutual information: 0.93, 95%-CI: 0.90–98).

Both the random forest classification and community detection analyses were repeated in the independent replication sample.

### Taxometric analysis

**Fig. 2:**
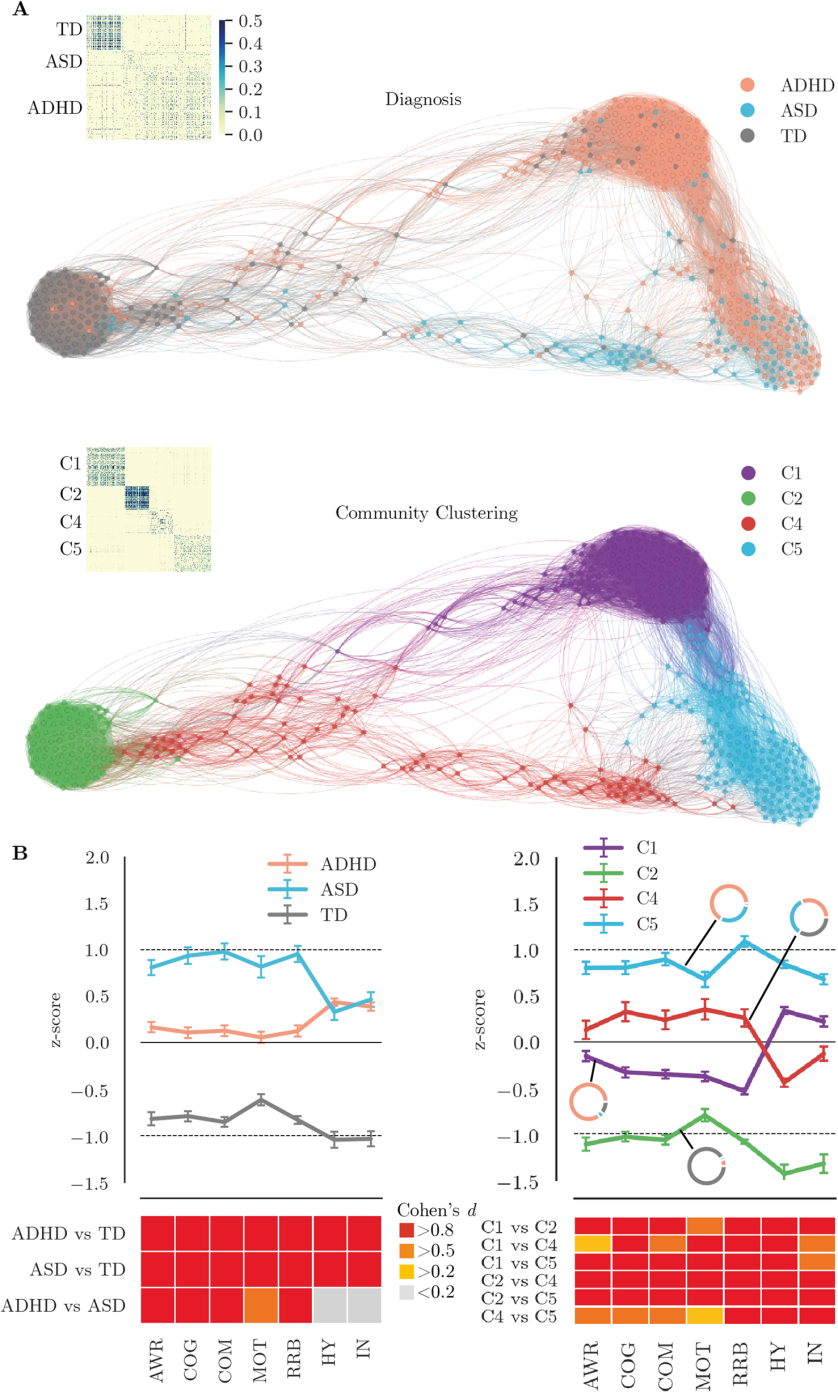
Profiles of diagnostic groups and groups identified through community clustering. **A** The proximity between participants according to the random forest classification is shown in Force Atlas layout [41] either colored according to the diagnostic group (top) or according to the groups identified through community detection (bottom). The smaller plots show the proximity matrix ordered according to either diagnostic or community detection labels. The figure illustrates the separation and overlap of the diagnostic groups as seen by the RFC algorithm. **B** Profiles of the groups according to diagnosis (left) or community detection (right). The lower part of the figure shows the effect size of comparisons between the groups. The circular plots in the right figure indicate the relative proportion of diagnoses within the groups identified through community detection. The error bars indicate one standard error around the mean. *AWR* social awareness, *COG* social cognition, *COM* social communication, *MOT* social motivation, *RRB* restricted interests and repetitive behaviors, *HY* hyperactivity/impulsivity, *IN* inattention

Because visual inspection of the community clustering solution in conjunction with the severity differences in the behavioral profiles of the clusters arguably (see Fig. 2) suggested a dimensional distribution of groups and scores, we conducted an additional exploratory analysis, which was not part of the pre-registration. In subsequent steps, we carried out taxometric analysis to assess if a dimensional or categorical account provided a better fit to the questionnaire data including either the diagnostic information or the clustering information. Taxometric analysis is based on bootstrap samples from the empirical data with equal sample size and number of indicators [72]. The data are generated under a dimensional or taxonic structural model. Subsequently, the fit of the observed data is evaluated. Prior to taxometric analysis, we assessed the suitability of the data for taxometric analysis defined by Ruscio, Ruscio, and Carney [71]. Tables with the corresponding *a priori* parameters can be found in the Supplementary Materials. We only included the main sample in the taxometric analysis, because the sample size of the replication sample was insufficient for this type of analysis. For the main sample, a solution with the three most important indicator variables as determined by the random forest classification (SWAN HY, SRS RRB, and SRS AWR) is presented in the main text below. The solution with three indicators is shown, because three indicators are the recommended minimum for taxometric analysis [71]. Solutions with two and four indicator variables can be found in the Supplementary Materials. As recommended in an authoritative review [71], we used a combination of fit indices for taxometric analysis that are implemented in the RTaxometrics package v2.3 [73]. The consensus result across the procedures is presented in the main text, i.e., the mean comparison curve fit index (CCFI). The CCFI indicates the relative fit of a categorical versus a dimensional model. CCFI values closer to 0 indicate stronger support for a dimensional model and values closer to 1 suggest better support for a categorical model. CCFI values between 0.4 and 0.6 are ambiguous as they indicate similar fit to both models [71].

### Statistical analysis

Group-wise comparisons were based on Welch-corrected *t* tests that account for differences in variance between the groups. Bonferroni correction was used to account for multiple comparisons and corrected *p* values are reported in the main text.

## Results

### Diagnostic groups show different profiles on questionnaires of social communication and ADHD symptoms

The diagnostic groups showed different profiles of scores on the SRS and SWAN questionnaires (analysis of variance (ANOVA)—group: F (2, 3017) = 801.8, *p* < 0.001; group x scale: F (12, 3016) = 10.7, *p* < 0.001). While both diagnostic groups showed higher scores compared to the TD group across all questionnaire scales (see Table 2 and Fig. 2), the ASD group scored higher on the SRS compared to the ADHD group. In contrast, there was no significant difference between the ADHD and ASD group for any of the SWAN subscales. Highly similar results were obtained in the replication sample (see Figure S3).

**Table 2:** Comparison of questionnaire profiles between the ADHD, ASD, and TD groups

	ADHD		ASD		TD		ADHD vs. TD			ASD vs. TD			ADHD vs. ASD		
	Mean	SE	Mean	SE	Mean	SE	*t*	df	*d*	*t*	df	*d*	*t*	df	*d*
AWR	0.17	0.056	0.83	0.084	− 0.81	0.073	− 10.66	266.04	− **1.18**	− 14.76	173.93	− **2.12**	− 6.55	149.33	− **0.83**
COG	0.10	0.058	0.97	0.093	− 0.78	0.057	− 10.80	329.26	− **1.14**	− 16.04	132.32	− **2.40**	− 7.92	140.95	− **1.02**
COM	0.13	0.056	1.00	0.087	− 0.85	0.055	− 12.45	328.52	− **1.32**	− 18.00	136.23	− **2.68**	− 8.40	145.44	− **1.08**
MOT	0.04	0.060	0.85	0.117	− 0.60	0.063	− 7.42	313.71	− **0.79**	− 10.92	120.30	− **1.66**	− 6.15	118.33	− **0.84**
RRB	0.11	0.059	1.02	0.091	− 0.82	0.044	− 12.51	354.97	− **1.28**	− 18.17	112.51	− **2.78**	− 8.40	146.28	− **1.08**
HY	0.44	0.040	0.35	0.087	− 1.02	0.086	− 15.40	181.81	− **1.81**	− 11.21	188.34	− **1.58**	0.98	111.12	0.14
IN	0.39	0.044	0.49	0.081	− 1.02	0.082	− 15.17	198.00	− **1.76**	− 13.11	190.97	− **1.85**	− 1.05	124.55	− 0.14

Significant differences are shown in bold print (*p* < 0.05)

*d* Cohen’s *d*, *df* degrees of freedom, *SE* standard error, *AWR* social awareness, *COG* social cognition, *COM* social communication, *MOT* social motivation, *RRB* restricted interests and repetitive behaviors, *HY* hyperactivity/impulsivity, *IN* inattention

### Random forest classification can predict diagnostic groups based on questionnaire data with some accuracy

Our results indicated that the optimal classification accuracy was achieved at a tree depth of two, i.e., two questionnaire scales were sufficient to discriminate the groups. Cross-validation supported that a tree depth of two was optimal for classification. At this depth, the accuracy of the model for the training set was 87% (CI = 83.03–90.44, κ = 0.79, McNemar’s *p* value = 4.6e^−8^) and 72% for the test set (CI = 62.76–80.17%, κ = 0.56, McNemar’s *p* value = 0.037; f1-score: 0.72, precision: 0.77, recall: 0.79). Sensitivity and specificity of the model indicated that diagnostic groups could be distinguished (see Table 3, ADHD: 0.67/0.84; ASD: 0.68/0.78; TD: 0.83/0.96 [sensitivity/specificity]). The most important scales for classification were SWAN HY (important as indicated by the percentage of trees that used the variable to split for classification: 100%) and SRS RRB (77.27%), followed by SRS AWR (63.37%), SRS COG (62.65%), SRS COM (57.20%), SRS IN (29.17), and SRS MOT (0.00%). The accuracy of the classification model was similar when applied to the independent replication sample (overall accuracy: 76%, ADHD: 0.69/0.84, ASD: 0.68/0.94, TD: 0.94/0.85 [sensitivity/specificity]).

**Table 3:** Confusion matrix for the held-out test data (outer loop)

Pred Ref	ADHD	ASD	TD
ADHD	41	6	2
ASD	17	13	3
TD	3	0	26

Rows indicate the predicted (Pred.) diagnostic group; columns indicate the actual diagnostic group (Reference [Ref.])

When excluding comorbid cases (ADHD with a diagnosis of ASD), the random forest classification reached an accuracy of 94.41% (*n* = 340, CI: 91.41–96.61%) in the training set and 71.17% (*n* = 111, CI: 61.81–79.37%; f1-score: 0.787, precision: 0.787, recall: 0.787) in the held-out test set. The specificity and sensitivity were acceptable for all classes (sensitivity/specificity, ADHD: 0.77/0.74; ASD: 0.63/0.82; TD: 0.65/0.98). Without the cases with a dual diagnosis, the SRS RRB seemed less important. The most important scales for classification were SWAN HY (100%) followed by SRS COM (76.59%), SRS AWR (72.65%), SRS COG (71.68%), SRS RRB (58.53%), SWAN IN (42.47%), and SRS MOT (0.00%).

### Community detection identifies subgroups that cross-diagnostic boundaries

The community solution consisted of five groups with four large groups (see Fig. 2, C1: *n* = 141 [31.26%], C2: *n* = 86 [19.7%], C4: *n* = 85 [18.85%], C5: *n* = 136 [31.16%]) and one small group^2^ (C3: *n* = 3 [0.67%]). The community detection algorithm converged at a stable solution that showed a good separation between the identified groups (*Q* = 0.92). The four large groups showed different profiles of questionnaire scores (ANOVA: group—*F* (2, 3108) = 738.01, *p* < 0.001; group x scale: *F* (18, 3109) = 63.49, *p* < 0.001, see Fig. 2 and Table 4). One group (C2: low symptoms) scored around 1 standard deviation (SD) below the other groups across all questionnaire scales and mostly contained children without a diagnosis (TD: 79 [85.11%], ADHD: 5 [10.64%], ASD: 2 [4.26%], comparison of proportions to the whole sample: *χ*^2^ = 288.10, *p* < 0.001). A second group (C5: high symptoms) had scores around 1 SD above the mean and consisted of two-thirds of children with ADHD and one-third of children with ASD (TD: 3 [2.92%], ADHD: 92 [67.15%], ASD: 41 [29.93%], *χ*^2^ = 226.05, *p* < 0.001). The other groups had contrasting symptom profiles. One group (C1: SWAN↑) showed low symptoms on the SRS scales, but high symptoms on the SWAN scales and consisted mostly of children with ADHD (TD: 15 [10.64%], ADHD: 120 [81.11%], ASD: 6 [5.26%]). Another group (C4: SRS↑) showed elevated symptoms on the SRS scales with lower ratings on the SWAN scales and consisted to equal proportion of children from all diagnostic categories (TD: 29 [34.12%], ADHD: 27 [31.76%], ASD: 29 [34.12%]).

**Table 4:** Comparison of questionnaire profiles between the community clustering-defined groups

	C1 (SWAN↑)		C2 (low symp)		C4 (SRS↑)		C5 (high symp)	
	Mean	SE	Mean	SE	Mean	SE	Mean	SE
AWR	− 1.11	0.068	− 0.15	0.055	− 1.11	0.068	0.80	0.069
COG	− 1.03	0.053	− 0.33	0.055	− 1.03	0.053	0.81	0.070
COM	− 1.07	0.052	− 0.35	0.047	− 1.07	0.052	0.90	0.067
MOT	− 0.81	0.067	− 0.38	0.053	− 0.8	0.067	0.68	0.083
RRB	− 1.08	0.018	− 0.53	0.033	− 1.08	0.018	1.09	0.055
HY	− 1.43	0.098	0.34	0.039	− 1.43	0.098	0.84	0.040
IN	− 1.32	0.098	0.22	0.055	− 1.32	0.098	0.68	0.055

	C1 vs. C2			C1 vs. C4			C1 vs. C5		
	*t*	df	*d*	*t*	df	*d*	*t*	df	*d*
AWR	− 10.92	183.37	− **1.50**	− 10.43	150.88	− **1.61**	− 19.73	209.51	− **2.66**
COG	− 9.19	216.36	− **1.22**	− 11.94	126.56	− **1.84**	− 20.97	219.31	− **2.74**
COM	− 10.24	199.40	− **1.38**	− 11.40	124.77	− **1.76**	− 23.08	219.76	− **3.02**
MOT	− 4.95	182.05	− **0.68**	− 8.89	137.81	− **1.37**	− 13.87	219.97	− **1.83**
RRB	− 14.67	207.41	− **1.84**	− 14.35	90.62	− **2.21**	− 37.63	163.19	− **4.67**
HY	− 16.74	111.86	− **2.46**	− 9.18	118.14	− **1.41**	− 21.41	113.89	− **3.15**
IN	− 13.78	138.47	− **1.97**	− 9.51	161.87	− **1.46**	− 17.86	138.46	− **2.56**

	C2 vs. C4			C2 vs. C5			C4 vs. C5		
	*t*	df	*d*	*t*	df	*d*	*t*	df	*d*
AWR	− 2.56	137.27	− 0.37	− 10.90	259.47	− **1.32**	− 5.64	163.70	− **0.79**
COG	− 5.72	134.73	− **0.82**	− 12.74	258.47	− **1.54**	− 3.90	161.51	− **0.55**
COM	− 5.26	119.82	− **0.77**	− 15.23	241.72	− **1.84**	− 5.39	155.32	− **0.76**
MOT	− 5.93	122.75	− **0.86**	− 10.72	230.38	− **1.30**	− 2.34	170.97	− 0.33
RRB	− 8.15	105.72	**− 1.21**	− 25.47	220.97	− **3.09**	− 7.78	143.59	− **1.11**
HY	13.23	193.68	**1.80**	− 9.07	274.15	− **1.09**	− 21.47	196.52	− **2.93**
IN	3.66	162.57	**0.51**	− 5.93	274.88	− **0.71**	− 8.45	162.18	− **1.19**

Significant differences are shown in bold print

*d* Cohen’s d, *df* degrees of freedom, *SE* standard error, *AWR* social awareness, *COG* social cognition, *COM* social communication, *MOT* social motivation, *RRB* restricted interests and repetitive behaviors, *HY* hyperactivity/impulsivity, *IN* inattention

The different groups were associated with differences in demographics and comorbid profiles: children in the cluster with higher SRS scores (C4) were slightly older compared to the rest of the sample, and there were more females in the cluster with low symptoms (C2) and more males in the cluster with high symptoms (C5). The other clusters did not deviate in sex ratio or age from the rest of the sample (see Table 5). Furthermore, the cluster with low symptoms (C2) and the cluster with relatively high SWAN scores (C1) contained fewer cases with a dual diagnosis of ASD and ADHD than would be expected given the proportion observed across the whole sample (see Table 5). In contrast, the cluster with high symptoms (C5) and the cluster with high SRS scores (C4) contained more ADHD-ASD comorbid cases than expected (see Table 5).

**Table 5:** Comparison of demographic information between the clusters

	C1 (SWAN↑)	C2 (low sym)	C4 (SRS↑)	C5 (high sym)
*N*	141	86	85	136
Male [%]	110 [78.72%]	45 [52.33%]	60 [70.59%]	113 [83.09%]
Stat	χ^2^ = 2.05, *p* = 0.152	**χ**^**2**^** = 19.55, *p* < 0.001**	χ^2^ = 0.34, *p* = 0.559	**χ**^**2**^** = 6.55, *p* = 0.011**
Age [mean ± SE]	9.34 ± 0.144	9.47 ± 0.161	9.72 ± 0.186	9.22 ± 0.133
Stat	*t* (254.21) = − 0.44	*t* (139.96) = 0.5	*t* (120.23) = 1.98	*t* (272.61) = − 1.54
	*p* = 0.659	*p* = 0.617	*p* = 0.05	*p* = 0.124
*n* comor. [%]	8 [5.67%]	0 [0.00%]	24 [28.24%]	60 [44.12%]
Stat	***χ***^**2**^** = 19.66, *p* < 0.001**	***χ***^**2**^** = 22.64, *p* < 0.001**	*χ*^2^ = 2.82, *p* = 0.093	***χ***^**2**^** = 44.66, *p* < 0.001**

For the statistical analysis, groups were compared to the frequencies observed across the whole sample regarding sex and comorbidity, and to the rest of the sample regarding age. Results shown in bold are significant at *p* < 0.05

The small cluster of three individuals (C3) contained one girl from the TD group, and two boys with both an ADHD diagnosis and an ASD diagnosis (ages between 7 and 9 years). This cluster was characterized by low social awareness ratings (AWR) and a high score for inattention (IN).

Community clustering in the replication sample produced clusters that were highly similar to the clusters identified in the main sample. Community clustering converged on a stable solution with three groups (*Q* = 0.93, see Figure S3). The first cluster mostly contained participants with a diagnosis of ADHD (CR1, *N* = 80 [36.53%]; ADHD: *n* = 73 [91.25%], ASD: *n* = 2 [2.5%], and TD: *n* = 5 [6.25%]). The second cluster mostly contained participants from the TD group (CR2, *N* = 64 [29.22%]; ADHD: *n* = 6 [9.38%], ASD: *n* = 1 [1.66%], and TD: *n* = 57 [89.06%]). The third cluster mostly contained participants with ASD diagnosis (CR3, *N* = 66 [88%]; ADHD: *n* = 8 [10.67%], ASD: *n* = 66 [88%], TD: *n* = 1 [1.33%]). The behavioral profiles of these groups were similar to the C1, C2, and C5 in the main sample. Similar to C1, CR1 showed was characterized by low scores on SRS scales and high scores on the SWAN scales. Like C2, CR2 showed low scores for all scales and CR3 showed high scores on all SRS scales and lower scores on the SRS scales like CR4.

### Taxometric analyses

Given these results, we next tested whether taxometric analyses would yield clear evidence in favor of either a dimensional or a categorical account of the differences between the diagnostic groups based on such discriminatory measures (SRS and SWAN). The comparative curve fit index (CCFI) can be used to investigate if a latent construct is dimensional (CCFI < 0.4) or categorical (> 0.6; [71] through comparison to simulated data in parallel analysis. Across different measures of curve fit, we found support for a dimensional distribution when including the typical and *both* atypical groups (mean = 0.36), and when including the ADHD and TD groups (mean = 0.35). The ASD-TD comparison was consistent with a categorical account (mean = 0.76). There was no strong support for either a categorical or a dimensional account for the comparison between ADHD and ASD (mean = 0.49, all based on 3 indicators; see Supplementary Materials for similar results obtained with 2 or 4 indicators). To test if the results were influenced by edge cases, we conducted a further taxometric analysis that only included cases that were assigned to one of the major clusters in the consensus community clustering analysis. Taxometric analysis indicated that all groups identified through community clustering were more compatible with a dimensional than a categorical account (see Table 6).

**Table 6:** Comparative curve fit index (CCFI) using three indicators for comparisons of the community clustering-defined groups and diagnostic groups

	L-mode	Mambac	Maxeig	Mean
C1 vs. C2	0.37	0.33	0.34	0.35
C1 vs. C4	0.37	0.33	0.35	0.35
C1 vs. C5	0.33	0.35	0.37	0.35
C2 vs. C4	0.33	0.35	0.39	0.36
C2 vs. C5	0.36	0.31	0.34	0.34
C4 vs. C5	0.37	0.34	0.36	0.36
(ADHD and ASD) vs. TD	0.29	0.39	0.40	0.36
ADHD vs. TD	0.21	0.45	0.39	0.35
ASD vs. TD	0.84	0.84	0.62	0.76
ADHD vs. ASD	0.70	0.48	0.30	0.49

CCFI values < 0.4 indicate a dimensional distribution; CCFI > 0.6 are more compatible with a categorical account

## Discussion

By adopting multiple analytical routes to subtyping, we investigate subgroups within a large cohort of typically and atypically developing children that either (a) represent a taxometric difference between ADHD and ASD or (b) indicate an underlying condition with ADHD and ASD as opposite ends of a dimension. Our results suggest that neither a *categorical* nor a *dimensional* characterization of the indicators used in this study (standard symptom scales) to define ADHD and ASD is more sensible than the other. In other words, the autistic and ADHD related behavioral traits as assessed in the current sample cannot, unambiguously, be characterized as either two separate clinical entities or as two opposite ends of a spectrum using taxometric analysis or community detection based on symptom scales. In contrast, we show that the difference between ADHD children and typical children in the current sample is *dimensional*, while the difference between ASD children and typical children can best be characterized as *categorical*. Whereas our results do not support the recent literature arguing for an underlying dimension that explains the frequent overlap of the two conditions, they do highlight the importance of studying ASD and ADHD in tandem, as has been suggested by many developmental researchers before (e.g., [27, 43, 69, 80]). We find that even with community detection techniques—focused on making detected groups most distinct—the results do not show separate groups of ADHD vs. ASD, but suggest that the behavioral symptom scales are not sufficient to fully distinguish the diagnostic groups. This is in line with the longstanding clinical practice in which clinicians are trained to not only base their diagnosis on these type of proxy reports of behavioral symptoms but take additional factors into account. Moreover, the community clustering results suggests subgroups with specific profiles that contain various mixtures of ADHD, ASD, and TD cases. This clustering solution may suggest relatively homogeneous subgroups that arguably provide a better characterization of the behavioral characteristics compared to the traditional diagnostic classifications (see [5, 7, 24] for similar arguments). However, even with these more homogeneous groups, the taxometric analysis did not fully support a categorical account.

The current study suggests that behavioral markers need to be established in cross-syndrome comparisons to distinguish markers that cut across disorder boundaries from markers that may be uniquely shared among a subset of individuals. First, our community detection and taxometric results suggest that ADHD and ASD cannot, unambiguously, be characterized as two separate clinical entities or as two opposite ends of a spectrum based on behavioral symptom scales. This is reflected in the moderate accuracy of the random forest classification that frequently confused ADHD and ASD cases, but could distinguish both groups from typical development with higher accuracy. This low accuracy for distinguishing ASD and ADHD was also observed when excluding cases with comorbid diagnoses. Furthermore, we applied community detection to test if a data-driven approach may provide a better grouping than the traditional diagnostic labels. While the algorithm identified clusters with more homogeneous behavioral profiles, taxometric analysis suggested that a dimensional account provided a better description of the data. However, the behavioral profiles of the clusters are not compatible with a single dimension of ADHD and ASD symptoms as has been previously suggested. While the identification of the cluster with the highest symptoms (C5) is in accordance with the overarching continuum hypothesis, under this hypothesis, we would also assume that high ADHD symptoms would be present in those with moderate ASD traits. While we did detect a cluster (C4) with somewhat lower ASD traits (C4), contrary to this hypothesis, these individuals did not show high ADHD symptoms.This is also reflected in the performance of the classification algorithm that confused around a third of ADHD and ASD cases, while classifying 80% of TD cases correctly. Moreover, as all identified clusters contain some combination of ADHD and ASD diagnoses, clinically, our results imply that screening for ADHD in individuals with an ASD diagnosis is imperative. Theoretically, our results underline taking a dimensional approach regarding the behavioral symptoms of ADHD and ASD that could advance knowledge about genetic, brain, and cognitive underpinnings of symptomatology. Dimensional analyses are useful when the association of clinical predictors with dimensional scores is constant for a relevant dimensional severity range [48]. To draw strong clinical policy-related conclusions such dimensionality first needs to be justified by demonstrating the absence of non-linear effects outside the clinical range that cause predictors to be significant for dimensional scores [48]. Moreover, we show that a completely dimensional view might be adequate for the relation between typical developing children and ADHD children, but does not do just to the complexity of the relationship between ADHD and ASD. Given the dimensional characterization that our results suggest regarding ADHD, clinicians will still need external criteria, such as impairment or suffering, to determine cut points on such dimensional measures that indicate the existence of impaired functioning.

Second, this study is the first to combine multiple analysis approaches with replication of cutting-edge subtyping and taxometric procedures to shed new light on one of the oldest psychometric issues in the field of atypical development research, i.e., the question of whether mental disorders should be thought of as discrete categories or as continua. In their review of psychometric modelling approaches, Borsboom and colleagues (2016) [11] note that the psychometric work related to this issue has not been able to put forward a systematic methodological procedure to investigate the kind vs. continua question. The authors suggest that this might be due to the limited range of hypotheses tested by common approaches as these procedures do not test the exhaustive hypotheses space of latent structures, but treat the potential answer as binary: evidence in favor of categorical distinction is treated as evidence against the hypothesis of a dimensional structure leaving no room for other (hybrid) possibilities, such as some alternative factor mixture models or network models do. We here proposed a combined framework of analytic steps that cover a wider hypothesis space from different methodological angles avoiding the abovementioned issue.

Third, previous autism research has suggested a taxon higher up the proposed gradient scale than DSM classification suggests, i.e., a ‘highly severe’ ASD subgroup [25]. We find that our taxometric results are ambiguous when performed comparing the ASD children with the ADHD children, instead of in all three groups. This ambiguity may be explained by the presence of a such a specific ASD subgroup. Also, the fact that ADHD diagnostic screening was part of the initial diagnostic assessment administered to the whole sample upon study entry but ASD screening was contingent on a clinician’s additional suspicion of ASD (see [1]) might explain the 70/30 division of ADHD/ASD diagnoses in the high symptom subgroup. Nevertheless, our taxometric analysis underlines the dimensional account of ADHD symptoms in typical children.

### Limitations

Despite several strong points of this study, including pre-registration, cutting-edge statistical techniques, a large sample size, and replication in an independent sample, several limitations should be considered when interpreting our results. First, it is important to note that the questionnaires used in this study are not specifically designed to distinguish diagnostic groups. Their purpose is to assess diagnostic severity and create symptom profiles—the latter allows us to investigate profiles that are more common in the ASD vs. the ADHD group. Although the validity of these assessments may vary with the clinical utility of the behavioral instruments used for this purpose, our results consolidate the findings of a recent grouping study based on core-domain symptomatology (as assessed by the SWAN rating scale) and cortical thickness, which suggested that ASD, ADHD, and OCD lie on a continuum with typical developmental profiles [50]. Second, it should be stressed that our analyses are based on validated ASD and ADHD symptom scales reflecting a wide range of behaviors and symptoms. Naturally, however, this focus does not cover all potential tributaries to ASD and ADHD phenotypes, such as neuropathological and genetic factors [74] or the effect of medication. Also, the SRS scale used in the current study mainly covers the ASD social domains, with only a few indicators of repetitive and restrictive behaviors and no assessment of the sensory sensitivities that often go along with ASD. The literature, however, suggests significant clinical difference between ADHD and ASD samples on this specific domain: reports of repetitive behaviors in ADHD are less frequent than reports of communicative and social difficulties [61]. Another large epidemiological study reports that repetitive and restrictive behaviors explain a substantial part of the co-occurrence of ASD and ADHD traits [65]. Future studies should, therefore, include extensive assessments of the whole range of symptoms.

Additionally, it should be noted that a taxometric approach to unveiling the latent structure of psychological conditions is not uncontroversial in psychometrics [11, 56]. We here explicitly accommodate all recent advances and recommendations by adopting taxometric procedures based on simulation [72] to deal with exceptions in its core assumptions (i.e., the assumption that categorical structures produce peaked covariance functions might not be true under certain conditions,[60]). Our results are, furthermore, based on (1) a large sample to make sure that sampling fluctuation has less impact [55] and (2) symptom scales with varying endorsement probabilities of their items [56].

Third, the current research on ADHD and ASD is highly skewed toward childhood, including this study. There are strong indications that the co-occurrence between ADHD and ASD is dependent on age (for review see [35]). For example, genetic research [77] indicated the shared dependency of ADHD and ASD symptoms on specific biological pathways but also notes that the impact of these pathways varies throughout development. Therefore, longitudinal research is warranted.

## Conclusion

In conclusion, this study supports those voices in the literature that are doubting the categorical differences between the consensus-based sets of ADHD symptoms and ASD symptoms; however, we also cannot state unambiguously that ADHD and ASD should be characterized as two opposite ends of a spectrum or as two separate clinical entities. In the long run, the statistical developments might result in a non-binary answer to the kind vs. continua question in psychiatry based on a novel way of conceptualizing non-linear transitions between different psychiatric conditions that follow from the complex interplay of their symptoms and the individual environment. For now, it is unambiguous that ADHD and ASD traits need to be studied in tandem.

### Supplementary Information

Below is the link to the electronic supplementary material.

Supplementary file1 (DOCX 19256 KB)

## Footnotes

1 Please note that multiple comorbid diagnoses were possible, i.e., the percentages are not additive.

2 Please note that we do not include this group (C3) in between-group comparisons, since there are only three people in that group.
