# Supplementary material for: Probing the overarching continuum theory: data-driven phenotypic clustering of children with ASD or ADHD
Source: Eur Child Adolesc Psychiatry. 2022 Jun 10;32(10):1909–23. doi: 10.1007/s00787-022-01986-9 (PMC10533623; doi:10.1007/s00787-022-01986-9)
Supplement: Supplementary file 1 — Supplementary file1 (DOCX 19256 KB) [file 787_2022_1986_MOESM1_ESM.docx]

## Correlation between questionnaire scales

**Table S1:** Correlation of questionnaire scales. The lower triangle shows correlations across all groups. The upper triangle shows correlations for the group of typically-developing children.

**Table S2:** Correlation of questionnaire scales. Upper triangle: ADHD group; Lower triangle: ASD group

**Classification using other machine learning algorithms**

In addition to the main analysis, we evaluated the influence of the particular algorithm and the cross-validation strategy. To this end, we ran classification with a l1- or l2-regularized support vector machine (SVM), ridge regression, or random forest classification. Regarding the cross-validation strategy, we implemented a stratified shuffle split, i.e. randomly selected sub-sets that retained the ratio of group in the full data and that served as a training and tests set, and a k-fold, i.e. fitting and testing the data in different splits of the data. We assessed the influence of the number of splits/folds (2, 3, 5, 10) and of the ratio of training vs test data (0.1-0.5). The results indicated similar performance of the k-fold and stratified shuffle split cross-validation strategies (see Figure S2 & S3). The random forest classification resulted in the best classification results (see Figure S2 & S3). The performance obtained with l1- or l2-regularised SVM was similar and the performance of ridge regression was worse. The influence of the number of folds was small for the stratified shuffle split.


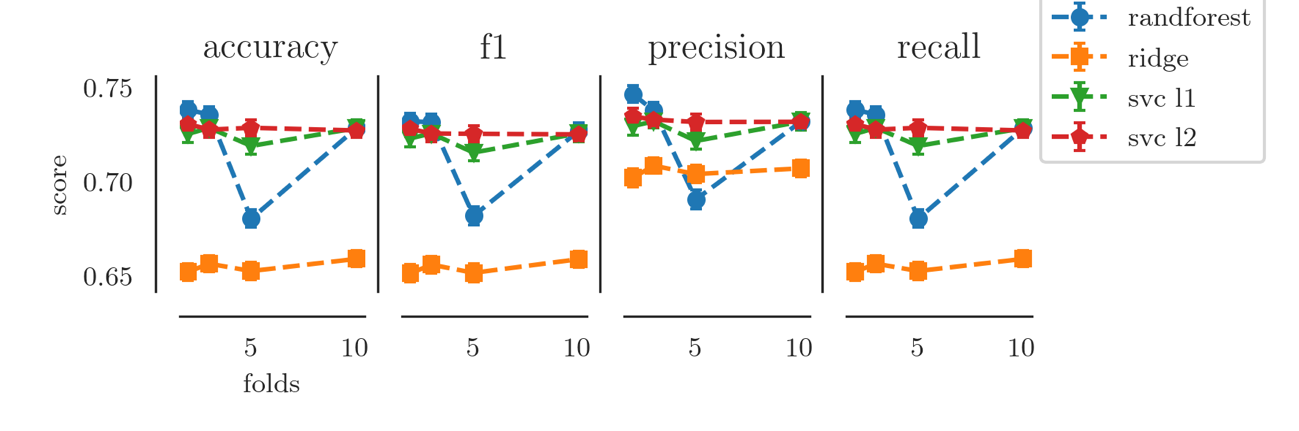


**Figure S1** Comparison of the classification performance using k-fold cross-validation. The influence of the number of folds was (x-axis) and machine learning algorithm (coloured lines) was assessed. The overall best performance was obtained at 2 folds using random forest classification. However, note the large difference in performance for 5-fold CV that may be driven by random sampling of some influential observations.


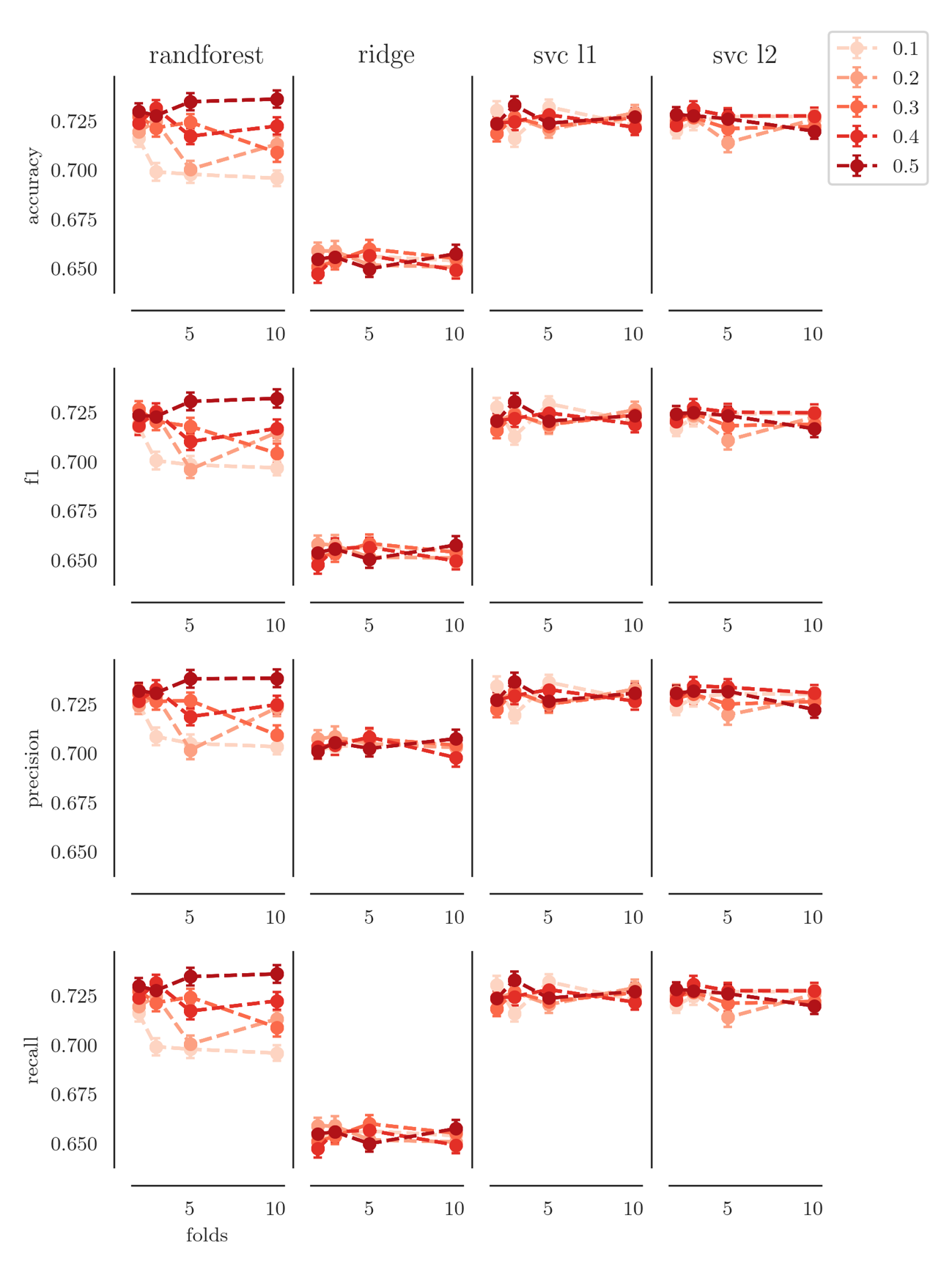


**Figure S2** Overview of the classification performance using a stratified shuffle split cross-validation strategy. The influence of the number of folds (x-axis), the ratio of training/test set (coloured lines) and the machine learning algorithm (columns) was assessed. The overall best performance was observed using random forest classification with 5 folds, and a training-test ratio of 0.5.

## Profiles of diagnostic groups in the replication sample


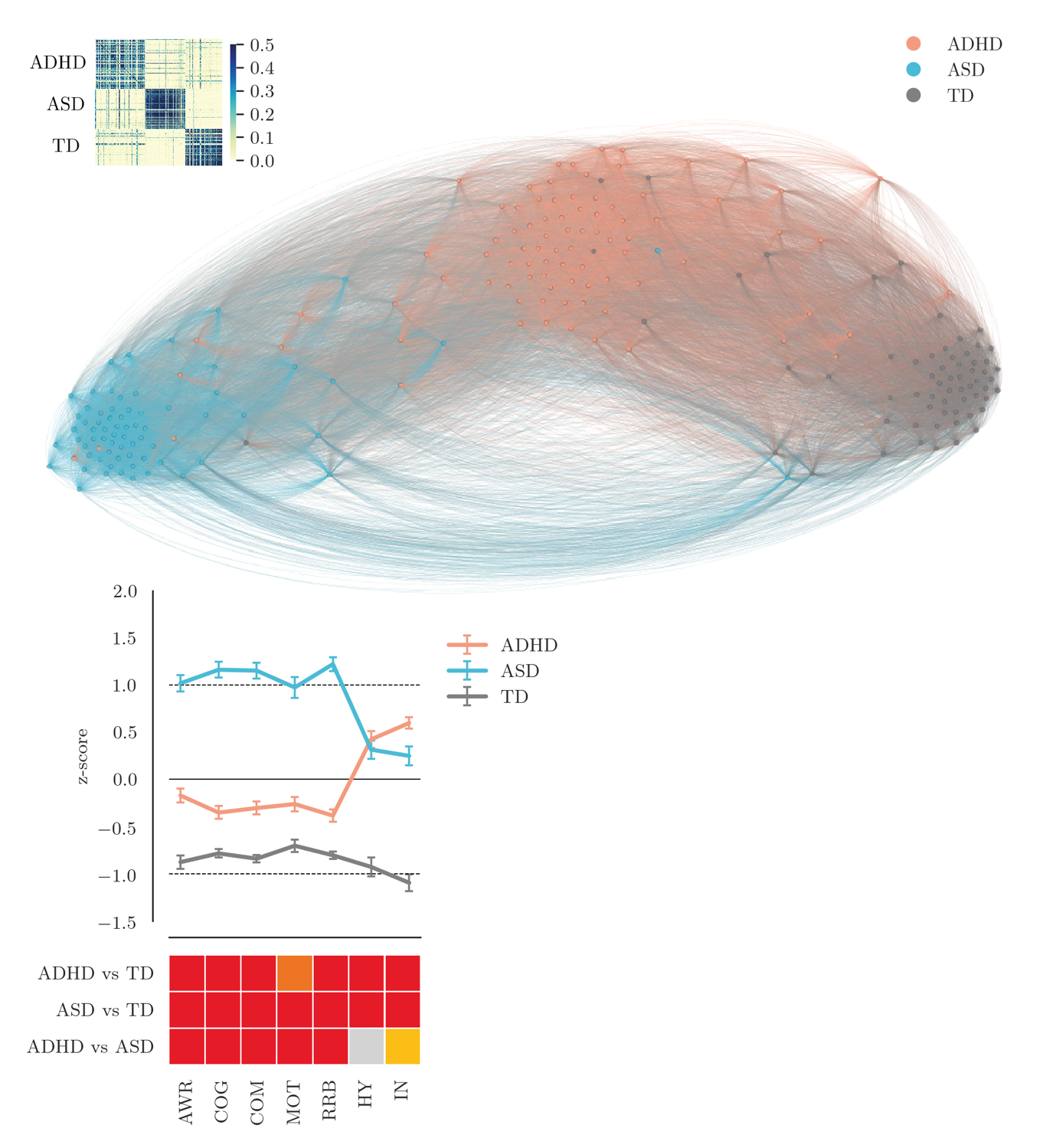


**Figure S3**: Profiles of diagnostic groups in the replication sample. Top: The proximity between participants according to the random forest classification is shown in Force Atlas layout (Jacomy, Venturini, Heymann, & Bastian, 2014) coloured according to the diagnostic group. The smaller plot shows the proximity matrix ordered according to diagnostic labels. The figure illustrates the separation and overlap of the diagnostic groups as seen by the RFC algorithm. Bottom: Profiles of the groups according to the diagnostic labels. The lower part of the figure shows the effect size of comparisons between the group. The error bars indicate one standard error around the mean. Abbreviations: AWR – Social Awareness, COG – Social Cognition, COM – Social Communication, MOT – Social Motivation, RRB – Restricted Interests and Repetitive Behaviours, HY – Hyperactivity/Impulsivity, IN – Inattention.

## Data checks for the taxometric analysis

The data were assessed for their suitability to perform taxometric analysis using the DataCheck function of the RTaxometrics v2.3 package. The taxon base rate (*p*) was set according to the size of the smaller of the diagnostic groups. The correlation value (*r*) was above the recommended cut-off for the comparison between ASD and TD. However, the value was close to the threshold for the analysis using 3 indicators (recommended: r<0.3). Further, the other indicators were within the recommended range. Ruscio, Ruscio & Carey state that “if […] data are at the borderline with regard to some criteria [sic] but acceptable on others, there may be relatively little risk in performing taxometric analysis so long as parallel analysis of comparison data is used…” (Ruscio, Ruscio, & Carey, 2011). Because the current analysis meets this description, the taxometric analysis should be valid despite the high correlation between indicators for the ASD vs TD comparison.

**Table S3:** Data checks for the taxometric analysis. The table lists recommended indices that test the suitability of the data for taxometric analysis. The metrics are: Cohen’s d – effect size of the comparison between putative taxons; n – the size of smaller putative taxon; p – the taxon base rate; r – the average correlation between the indicators. Indicators printed in bold are outside of the recommended range for taxometric analysis (Ruscio, Ruscio, & Carney, 2011).

## Taxometric analysis

**Table S4:** The table lists the Curve Comparison Fit Index (CCFI) for metrics used with 2 indicators and 3-4 indicators. Values <0.4 are more compatible with a dimensional account, value >0.6 support a categorical account. Values between >0.4 and <0.6 are ambiguous. The consensus between methods, i.e. the mean CCFI values, is considered most informative.

##
